# Photothermal stability of biologically and chemically synthesized gold nanoprisms

**DOI:** 10.1007/s11051-017-4027-z

**Published:** 2017-09-24

**Authors:** Magdalena Klekotko, Joanna Olesiak-Banska, Katarzyna Matczyszyn

**Affiliations:** Advanced Materials Engineering and Modelling Group, Faculty of Chemistry, Wroclaw University of Science and Technology, Wyb. Wyspiańskiego 27, 50-370 Wrocław, Poland

**Keywords:** Gold nanoparticles, Biological and chemical synthesis, Photostability, Femtosecond laser irradiation

## Abstract

We report here the influence of the irradiation with femtosecond laser pulses on the gold nanoprisms synthesized using biological and chemical methods. For the bio-mediated growth, we used plant extract as a source of reducing, structure-directing, and stabilizing agents, while for the chemical method, we applied three-step protocol, involving chemicals commonly used in the synthesis of nanostructures. Exposition of the nanostructures to the laser beam causes morphological changes, which affect their extinction spectra. These modifications were followed using absorption spectroscopy and transmission electron microscopy. The observed effects depend on the applied laser power and excitation wavelength. Under resonance conditions, rounding of the tips of triangular nanoparticles and transformation towards more stable, spherical form were noticed. These changes were faster under higher laser power. Such shape modifications were weaker under off-resonance conditions. Moreover, chemically synthesized gold nanoprisms were less susceptible to the morphological changes than those obtained using plant extract; however, their colloidal stability was disrupted by long-time irradiation.

**Graphical abstract:**
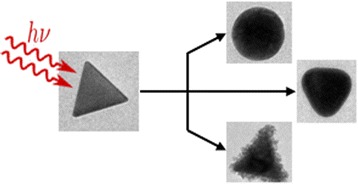
ᅟ

ᅟ

## Introduction

Intensive interest in gold nanoparticles (GNPs), caused by their wide range of applications in many fields of physics, chemistry, and biology, resulted in the development of numerous methods of the fabrication of gold nanostructures with various sizes and shapes (e.g., spheres (Liu et al. 2015; Polte et al. 2010; Zhou et al. 2009), rods (Chang et al. 1999; Sau and Murphy 2004; van der Zande et al. 2000), wires (Liu et al. 2006), prisms (Scarabelli et al. 2014), cubes (Thiele et al. 2016), and stars (Yuan et al. 2012)). Many applications of such nanoparticles, including two-photon microscopic imaging (Olesiak-Banska et al. 2013; Wang et al. 2005), photothermal therapy (Huang et al. 2007), and biosensing (Zhao et al. 2016), involve laser irradiation of the sample. On the other hand, laser beam may be applied for the post-synthetic modification of the GNP morphology (Gordel et al. 2014; Huang et al. 2005; Link et al. 2000). Therefore, attention should be paid to details of the nanoparticle-laser interactions, and, indeed, various studies have been reported on the photothermal stability of the GNPs, taking into account the shape of the nanostructures, stabilizing agents, laser pulse duration, excitation wavelength, laser power, and irradiation time (Gonzalez-Rubio et al. 2016; Hashimoto et al. 2012; Pustovalov 2016). In most cases, treatment of nanoparticles with the laser beam has been found to change their morphology towards more spherical form, which is the most favored particle shape from thermodynamic point of view (Parinda et al. 2014; Warshavski et al. 2011; Zijlstra et al. 2009).

In this paper, we present the comparison of the photothermal stability of gold nanoprisms synthesized using biological and chemical methods. The bio-mediated synthesis used here applied a plant extract as reducing, structure-directing, and stabilizing agent (Klekotko et al. 2015), while the chemical protocol included reduction of gold ions with sodium borohydride (formation of the seeds) and ascorbic acid (growth of the GNPs) and stabilization of the formed nanoprisms with CTAC (cetyltrimethylammonium chloride) (Scarabelli et al. 2014). Then, the prepared gold nanotriangles were exposed to the femtosecond laser pulses with the increased exposition time. The effect of the irradiation was monitored by recording the changes of the UV-Vis spectra of the GNPs after increased time intervals. The morphological modifications were analyzed by transmission electron microscopy (TEM).

## Materials and methods

### Reagents

Tetrachloroauric(III) acid (HAuCl_4_·3H_2_O), sodium borohydride(NaBH_4_), cetyltrimethylammonium chloride, sodium iodide (NaI), and L-ascorbic acid were obtained from Sigma-Aldrich and used as received. Dried, powdered leaves of *Mentha piperita* were purchased in a local market.

### Synthesis of gold nanoprisms

Biosynthesis of gold nanoparticles (B-GNPs) was carried out using water extract of mint (*Mentha piperita*) (Klekotko et al. 2015). Briefly, 250 μL of the plant extract (1 g of the mint leaves per 10 mL of water) was added to 5 mL of 1 mM HAuCl_4_ aqueous solution, followed by 24 h incubation in the dark, at 28 °C. After the reaction was completed, the obtained nanoparticles were centrifuged and the pellet was resuspended in distilled, deionized water. We assume, that such nanoparticles are stabilized with the specific parts of the proteins containing the functional groups, which bind preferentially to the surface of a gold core (for example, through thiol groups). The diversity of the obtained structures may be related to the complexity of the plant extract and occurrence of various reduction processes in the reaction mixture.

For the chemical synthesis of gold nanoprisms (Ch-GNPs), we applied the three-step method, reported by Liz-Marzán’s group (Scarabelli et al. 2014). The protocol includes seeds preparation and two growth steps. The seeds were prepared by addition of 25 μL of 0.05 M chloroauric acid solution to 4.7 mL of 0.1 M CTAC solution, followed by 300 μL of freshly prepared 0.01 M sodium borohydride. After 2 h, the seed solution was diluted 10× in 0.1 M CTAC solution. In the next step, 50 μL of the diluted seed solution was added to the 1st growth solution prepared by mixing 4 mL of distilled, deionized water along with 0.8 mL of 0.1 M CTAC solution, 20 μL of 0.05 M HAuCl_4_ solution, 7.5 μL of 0.01 M NaI solution, and 20 μL of 0.1 M ascorbic acid solution. Then, 1.6 mL of overgrown nanoparticles was added to the 2nd growth solution consisting of 20 mL of 0.05 M CTAC solution, 250 μL of 0.05 M HAuCl_4_ solution, 150 μL of 0.01 M NaI solution, and 200 μL of 0.1 M ascorbic acid solution. The obtained mixture was shaken for a few seconds and then left undisturbed for 1 h.

### Characterization of obtained nanoparticles

UV-Vis absorption spectroscopy and transmission electron microscopy (TEM) were applied to characterize the obtained nanoparticles. Spectroscopic measurements were performed using a JASCO V-670 spectrophotometer and 10-mm quartz cuvettes. Extinction spectra were recorded in the 400–1200 nm range. The microscopic observations were done using a FEI Tecnai G2 20 X-TWIN microscope. Samples for the TEM measurements were prepared on carbon-coated copper grids by putting a drop of the solution of the nanoparticles and evaporating the solvent.

### Laser-induced melting experiment

The melting experiment was conducted using a Quantronix Integra-C regenerative amplifier operating at 800 nm (repetition rate—1 kHz, pulse duration < 130 fs) and pumping a Quantronix-Palitra-FS BIBO crystal-based optical parametric amplifier which provided wavelength tunability. Polarizing wavelength separators and color glass filters were used to separate the required wavelengths, and the laser power was attenuated as needed with neutral-density filters. The solution of gold nanoprisms was poured into a quartz cuvette with a stirring bar inside. The sample was mixed on a magnetic stirrer (500 rpm) and irradiated by the laser beam (with 6-mm spot size) delivered directly from the setup, without any further concentration of the light on the lens. The B-GNPs were treated with the laser wavelength set at 800 nm, applying two laser powers: 920 mW (laser power density 3.25 W/cm^2^) and 30 mW (laser power density 0.11 W/cm^2^). The photothermal stability of the Ch-GNPs was examined under off-resonance conditions (laser wavelength of 800 nm, laser power of 920 mW, and laser power density of 3.25 W/cm^2^) and under resonance conditions (laser wavelength of 650 nm, laser power of 30 mW, and laser power density of 0.11 W/cm^2^). The melting of the nanoparticles was monitored by recording the temporal changes of the UV-Vis spectra. Reshaping of the GNPs was confirmed by TEM.

## Results and discussion

### Synthesis and characterization of gold nanoprisms

**Fig. 1:**
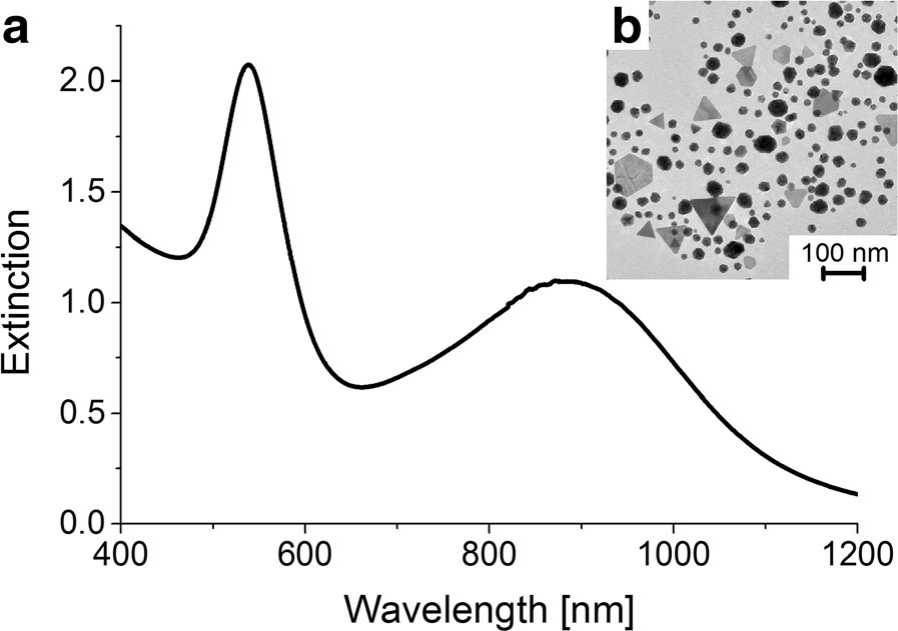
**a** UV-Vis spectrum. **b** TEM image of gold nanoparticles synthesized using mint extract

Change of the reaction mixture color during the synthesis indicated the formation of gold nanoparticles. UV–visible spectral analysis confirmed the presence of the GNPs in the solution. Extinction spectrum of the gold nanoparticles synthesized using mint extract (Fig. 1a) revealed two surface plasmon resonance (SPR) bands. The first peak is placed at 540 nm and corresponds to the spherical gold nanoparticles, while the second one, shifted towards longer wavelengths, indicates the presence of anisotropic shapes in the mixture. Transmission electron microscopic observations were performed to examine the morphology of the formed GNPs. TEM images show that the synthesis using mint extract results in the formation of polydisperse gold nanoparticles with various sizes and shapes (Fig. 1b). The UV-Vis spectral analysis and microscopic observations revealed that the biological synthesis of triangular nanoparticles is not as efficient as the chemical one. Despite that, in the images, we could clearly indicate this type of the GNPs. The statistical analysis of the TEM images allowed us to estimate the percentage of these particular structures, which account ~ 10% of the obtained mixture. The length of the edge of formed triangular nanoparticles varies from 30 up to 300 nm and the thickness is around 10 nm. The broad extinction band in the range of wavelengths from 700 to 1100 nm results mostly from the triangular nanoparticles with various sizes, which are the second largest group of the GNPs in the mixture. However, due to the presence of some other shapes of the GNPs, visible in the TEM images, we assumed, that this band corresponds to all anisotropic structures present in the solution.

**Fig. 2:**
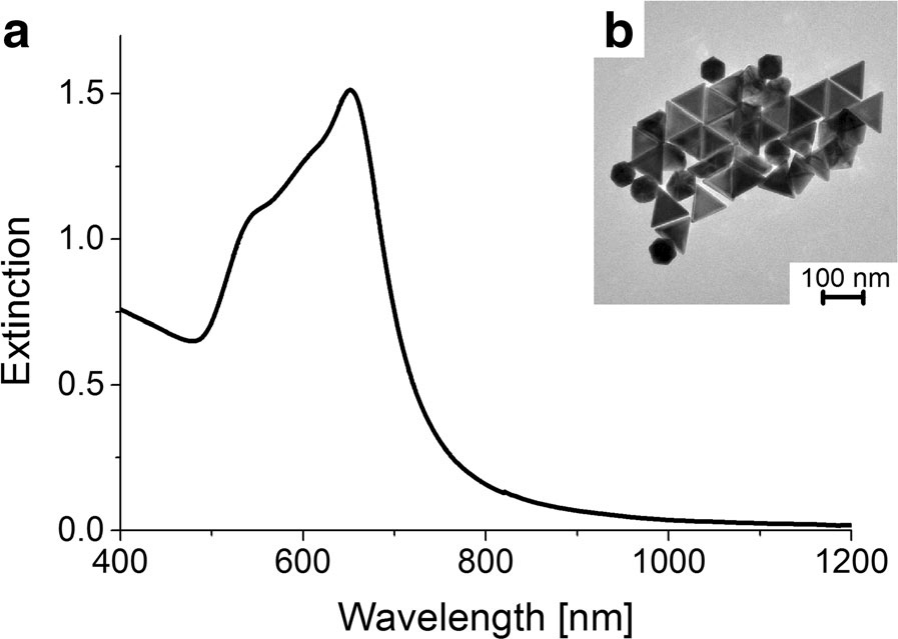
**a** UV-Vis spectrum. **b** TEM image of gold nanoprisms synthesized chemically

The UV-Vis spectrum of the chemical GNPs exhibits three, unseparated SPR bands (Fig. 2a). The signal corresponding to the gold nanoprisms (placed at 650 nm) is shifted towards shorter wavelengths, comparing to the biological ones. It may be related with the differences in the morphology of the formed nanoparticles (the Ch-GNPs are smaller in term of the edge length, but thicker than the B-GNPs). Furthermore, the peak is narrower, implying the formation of more uniform nanoprisms, which is also confirmed by TEM images, that show prismatic nanoparticles with the edge length of 85 ± 10 nm and the thickness of around 30 nm, with a tendency for self-organization (Fig. 2b). Moreover, the SPR band of the gold nanoprisms is stronger than the one from gold nanospheres (placed at 540 nm), which indicates higher concentration of triangular shapes. The obtained mixture contains up to 70% of prismatic nanoparticles.

### Laser-induced melting experiment

We compared the photothermal stability of biological and chemical gold nanoprisms treated with the femtosecond laser pulses. At first, the samples were exposed to the laser power of 920 mW at the wavelength of 800 nm (laser fluence of 3.2 mJ/cm^2^). We recorded the changes of the UV-Vis spectra of the samples with the increased exposition time.

**Fig. 3:**
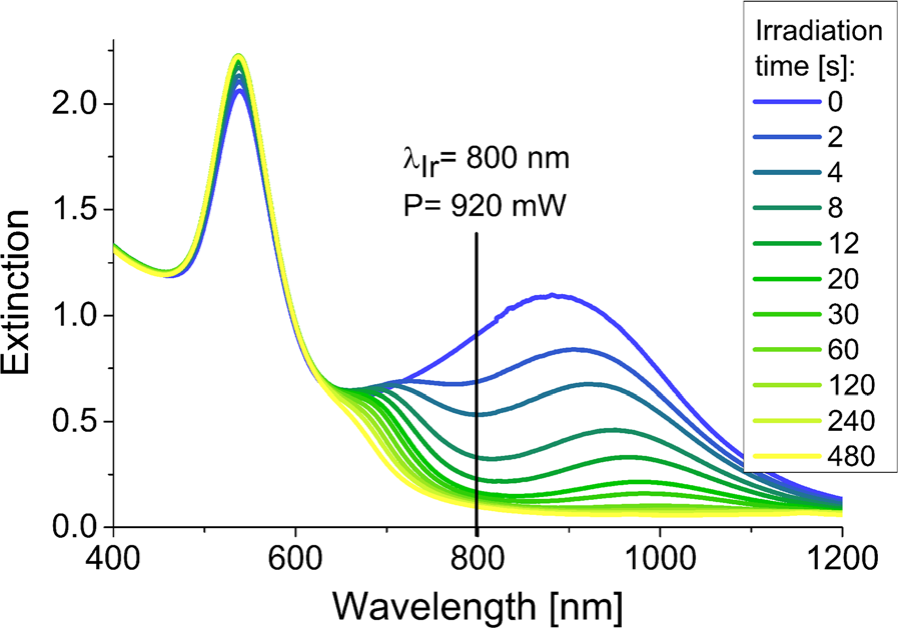
Changes of the UV-Vis spectra of the B-GNPs as a function of irradiation time (irradiation wavelength—800 nm, laser power—920 mW)

Irradiation of the GNPs synthesized using mint extract resulted in the rapid decrease of the extinction in the range of wavelengths corresponding to the anisotropic shapes and the slight increase of the SPR band coming from spherical nanoparticles (Fig. 3). In the beginning, we could observe the appearance of the two additional peaks placed on both sides of the irradiation wavelength. The first one, shifted towards shorter wavelengths, is related with the formation of partially melted structures with the rounded tips, but not fully converted to the spherical form (Lou et al. 2017). The second band, shifted towards longer wavelengths, may be connected with the occurrence of the bigger structures (present in the in the initial mixture or formed during the melting process), which absorb the incident light less efficiently (Huang et al. 2005). However, finally, all the anisotropic nanostructures were transformed to the spherical forms, which resulted in the disappearance of all signals except the one placed at 540 nm, corresponding to the nanospheres.

**Fig. 4:**
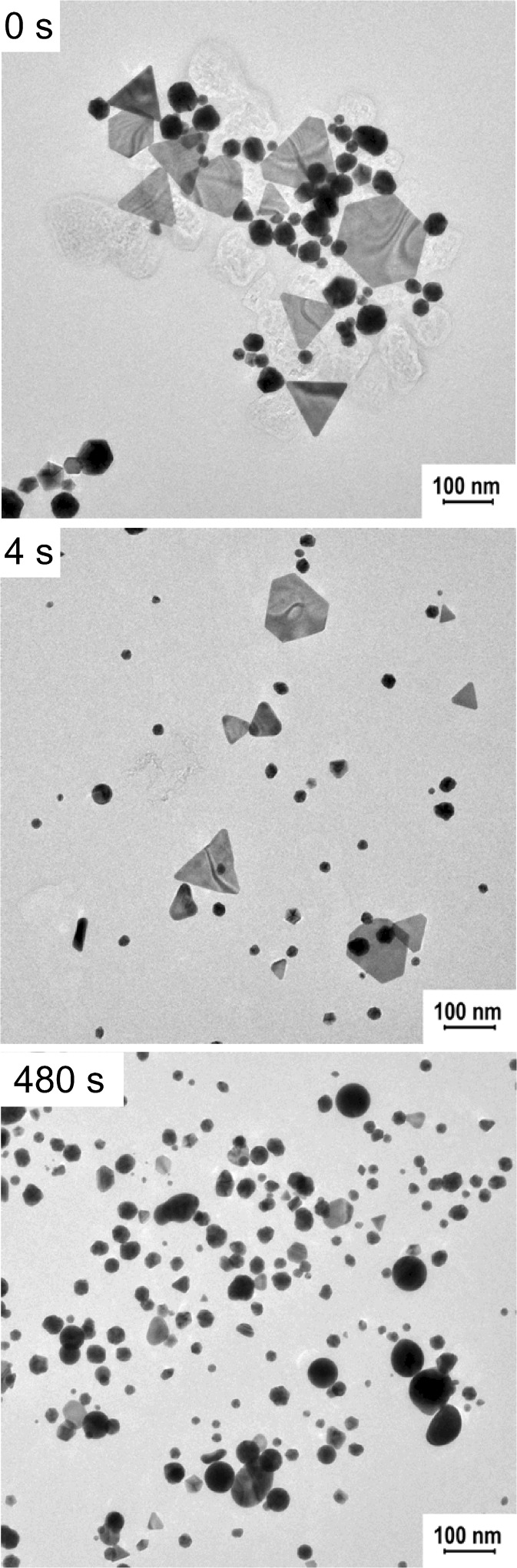
TEM images of the B-GNPs after laser irradiation (irradiation wavelength—800 nm, laser power—920 mW)

To examine the morphological changes, we selected two samples for the microscopic analysis and compared them with the control, non-irradiated sample (Fig. 4). After 4 s of irradiation, we could notice reshaping of the structures in the mixture and after 8 min, almost all triangular nanoparticles were melted.

**Fig. 5:**
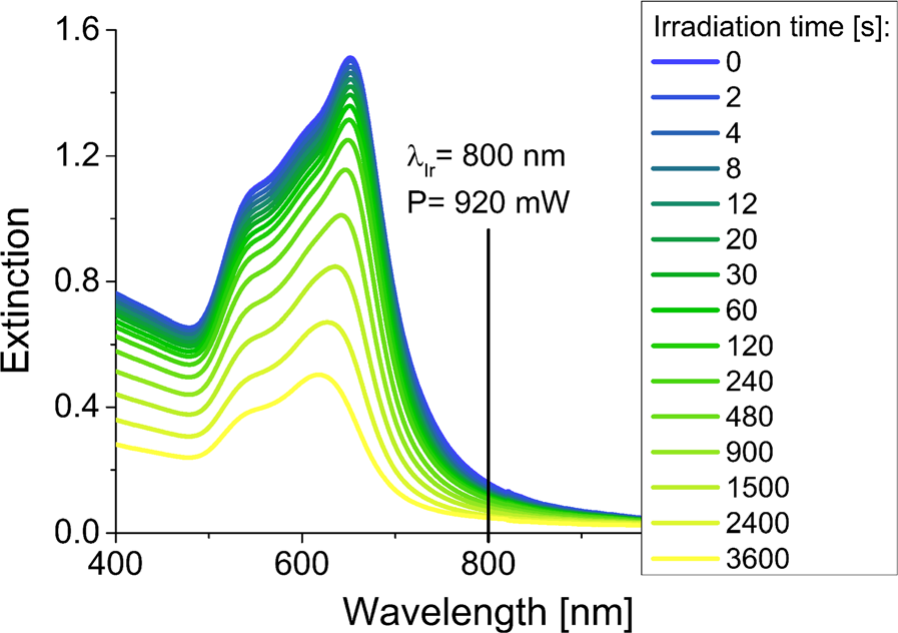
Changes of the UV-Vis spectra of the Ch-GNPs as a function of irradiation time (irradiation wavelength—800 nm, laser power—920 mW)

In the case of the GNPs synthesized chemically, the exposure of the sample for even 1 h did not cause the complete melting of the nanoprisms (Fig. 5). This can be related with the mismatch between the irradiation wavelength and the SPR band of the chemically synthesized gold nanoprisms. However, long-time irradiation caused the deposition of the nanostructures on the walls of the cuvette and disruption of further measurement.

**Fig. 6:**
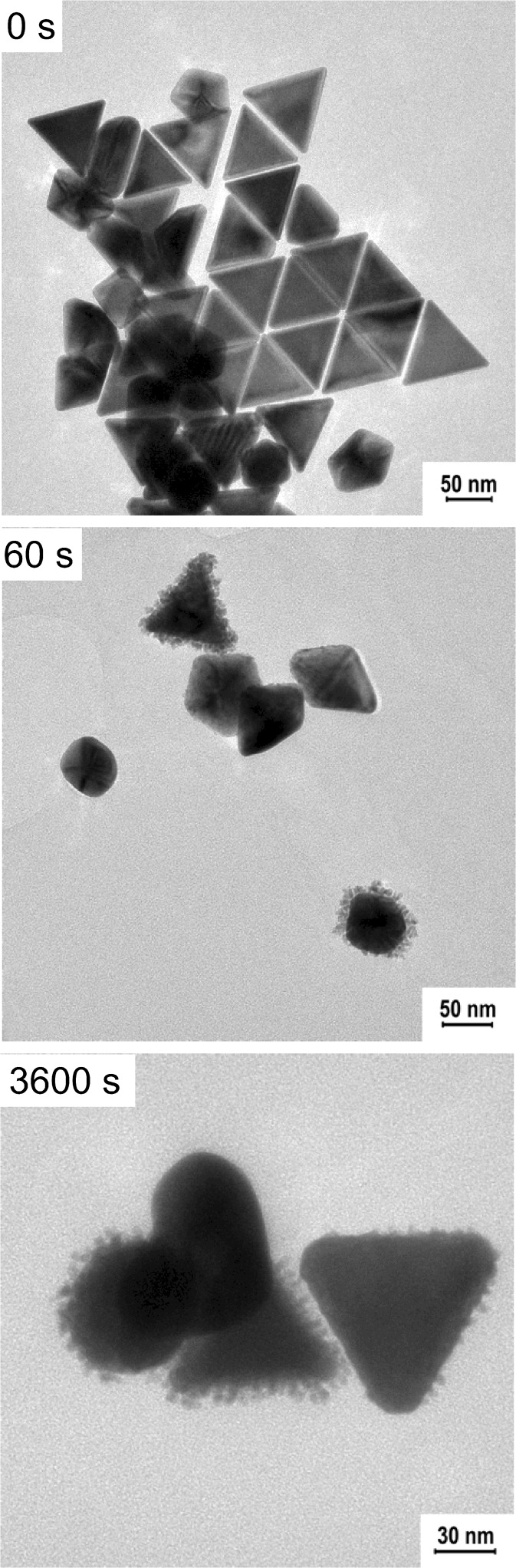
TEM images of the Ch-GNPs after laser irradiation (irradiation wavelength—800 nm, laser power—920 mW)

After 1 min of irradiation of the Ch-GNPs, we could notice some changes at the edges of the nanostructures, which may be related with the size reduction of the irradiated nanoparticles and formation of smaller ones, caused by the surface evaporation of the gold atoms (Liu et al. 2015). However, even after 1 h of irradiation, we still observed prismatic shapes in the mixture (Fig. 6).

**Fig. 7:**
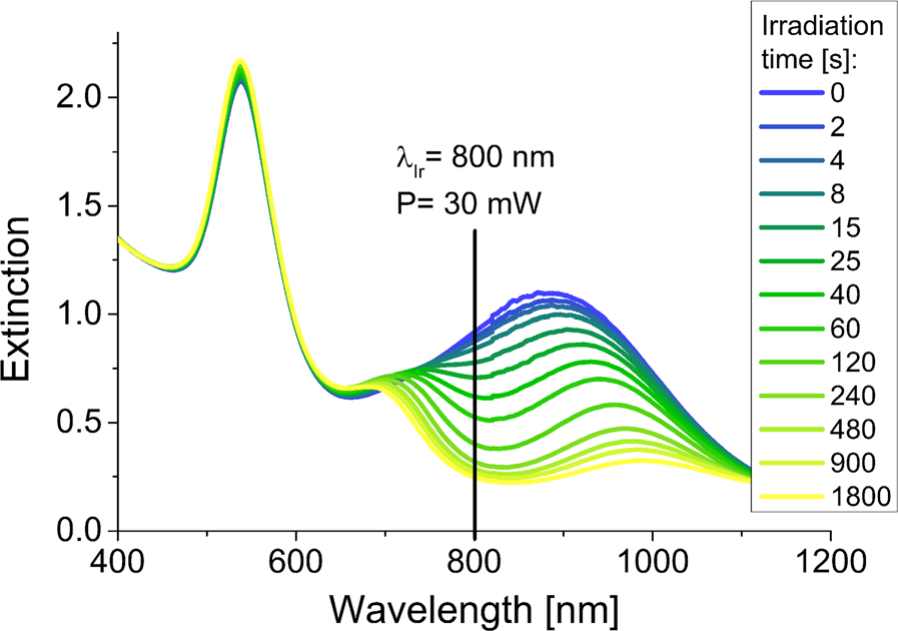
Changes of the UV-Vis spectra of the B-GNPs as a function of irradiation time (irradiation wavelength—800 nm, laser power—30 mW)

Subsequently, we compared the effects obtained using laser wavelengths placed within the SPR bands of both types of gold nanoprisms and with comparable laser power. For B-GNPs, we applied 800 nm wavelength with the laser power reduced to 30 mW (laser fluence of 0.11 mJ/cm^2^). Similarly to the previous results, we could observe the decrease of the extinction intensity at the wavelength corresponding to the irradiation wavelength (analogous to hole burning in inhomogeneously broadened bands) and appearance of two separated bands shifted into opposite directions (Fig. 7). However, the melting process was definitely slower and concerned mainly the nanoparticles, which absorbed the incident light efficiently. Irradiation of the mixture with decreased laser power did not affect newly formed populations of the nanoparticles as much as the high-power beam. Consequently, we did not observe the full melting of the anisotropic structures.

**Fig. 8:**
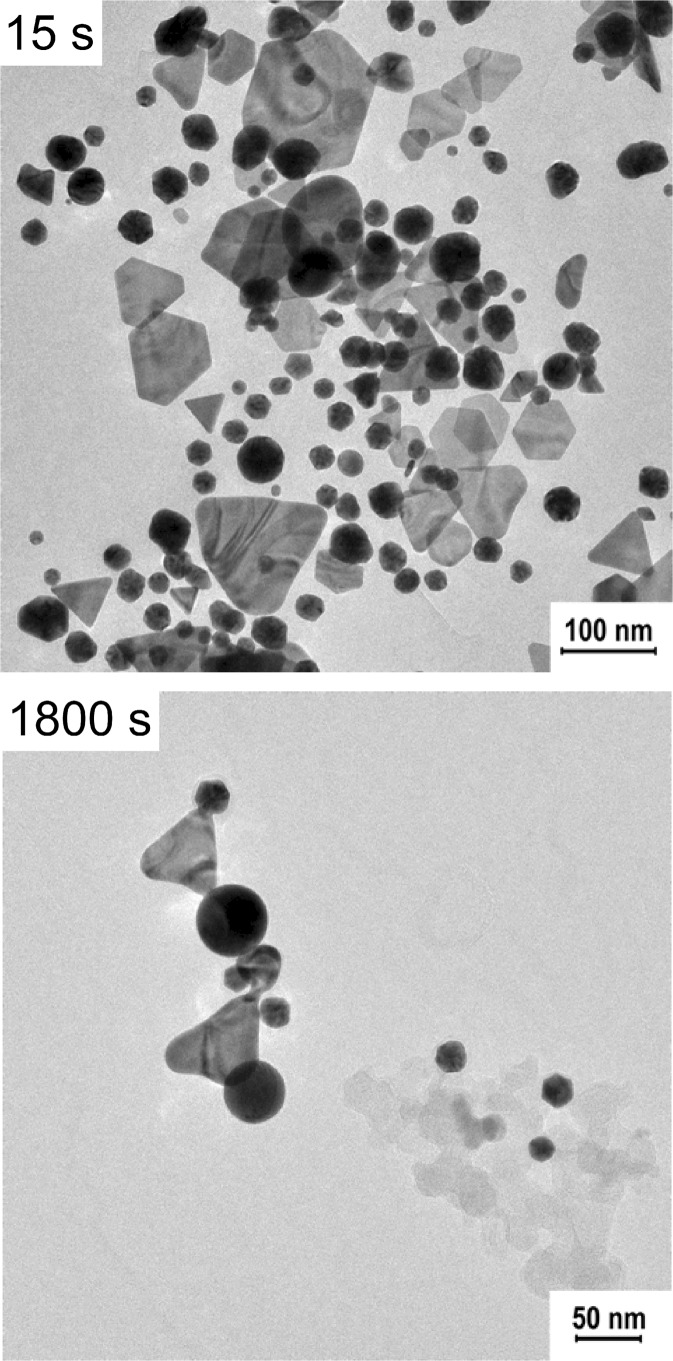
TEM images of the B-GNPs after laser irradiation (irradiation wavelength—800 nm, laser power—30 mW)

TEM images confirm the formation of melted spherical structures in the sample (Fig. 8). Nevertheless, after 30 min of irradiation there were still some triangular nanoparticles in the mixture.

**Fig. 9:**
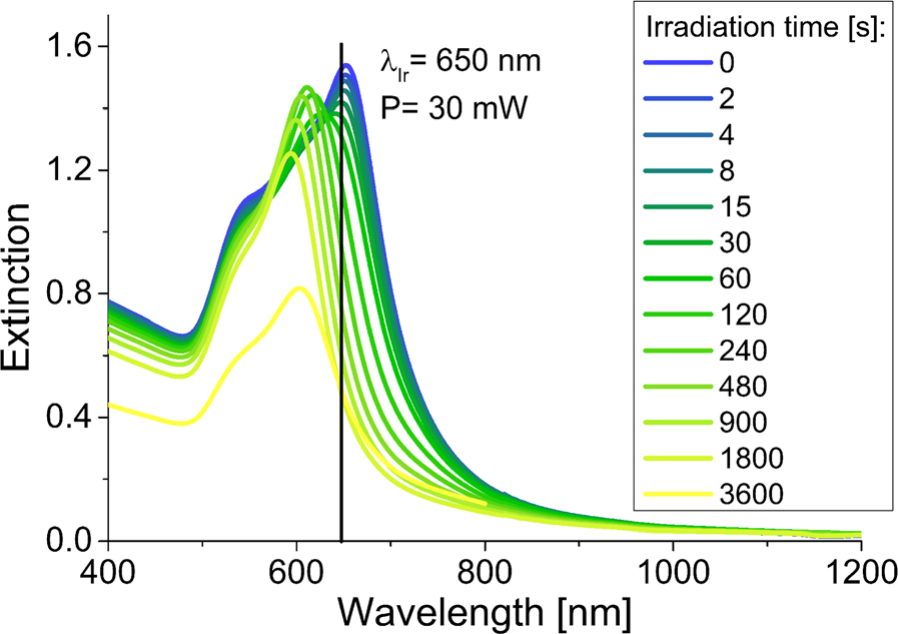
Changes of the UV-Vis spectra of the Ch-GNPs as a function of irradiation time (irradiation wavelength—650 nm, laser power—30 mW)

For the chemically synthesized gold nanoprisms, we applied the same laser power of 30 mW, but changed the irradiation wavelength to 650 nm. At first, we noticed a slight decrease of the extinction intensity of gold nanoprisms, but then the SPR band shifted towards shorter wavelengths and simultaneously the intensity increased (Fig. 9).

**Fig. 10:**
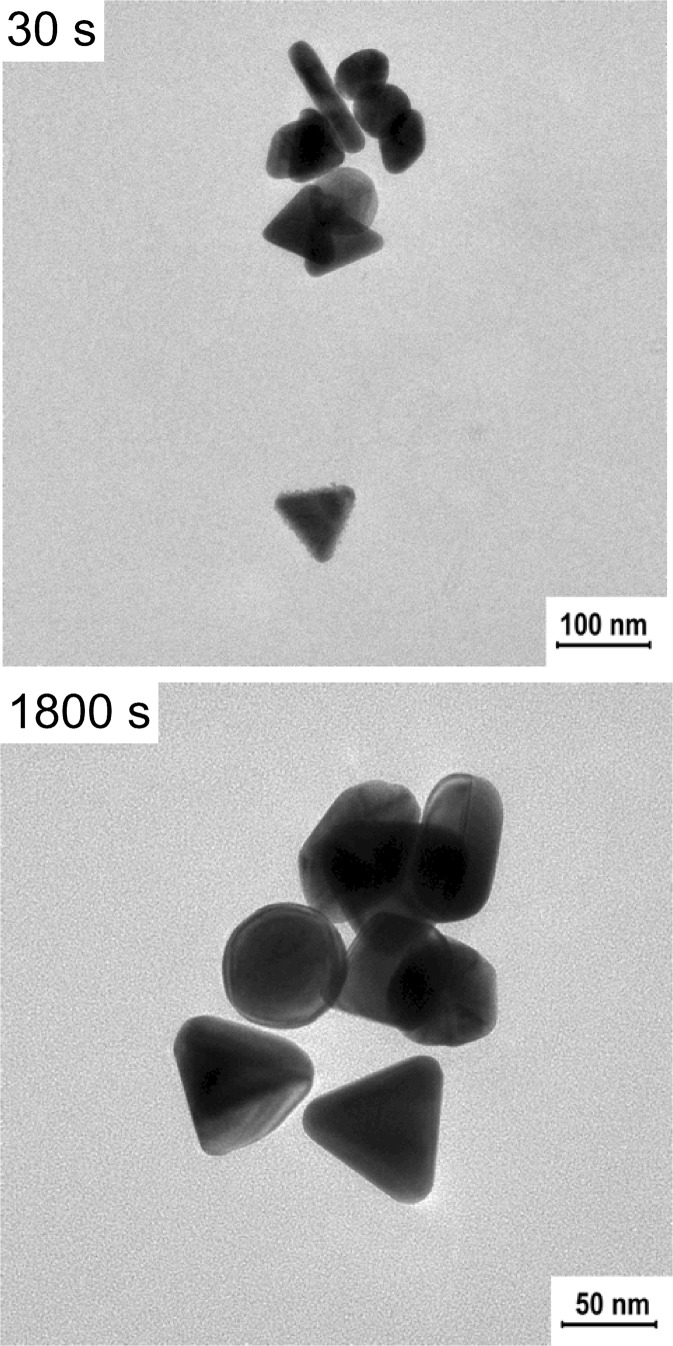
TEM images of the Ch-GNPs after laser irradiation (irradiation wavelength—650 nm, laser power—30 mW)

In that case, the incident light was highly absorbed by the prismatic nanoparticles, causing deformation of the tips and blue-shift of the plasmon band (Huang et al. 2005). The progressive melting of the nanoprisms led to the increase of the polydispersity of the nanoparticles in the mixture, which resulted in the decrease of the extinction intensity at 650 nm and broadening of the extinction band. Further irradiation of the solution promoted the formation of higher amount of the nanoparticles with the rounded tips from sharply ended nanoprisms, which was observed as further decrease of the extinction intensity at the wavelength corresponding to the nanoprisms, blue-shift of the extinction band, and increase of the extinction intensity at the range of wavelengths absorbed by the newly formed nanoparticles. Eventually, similar to the previous measurement, we observed the decrease in the total concentration of GNPs in the solution after long-time irradiation, caused by the deposition of the nanostructures on the walls of the cuvette. Microscopic observations revealed slight surface modifications of chemical gold nanoprisms caused by the irradiation (Fig. 10). In the sample exposed to the 30 min irradiation, we could distinguish some melted structures along with unmodified nanoprisms.

**Fig. 11:**
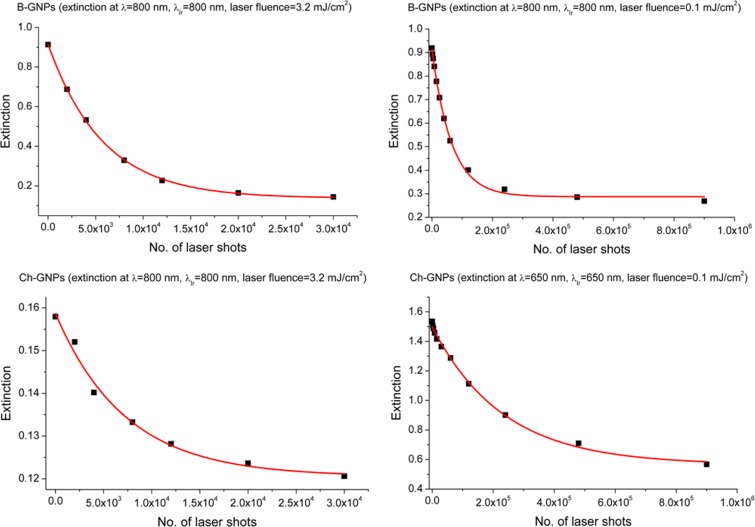
Changes of the extinction at the irradiation wavelength as a function of the laser shots, with the fitting to the first-order rate law. Fitting of the data enabled to calculate the No._1/e_ parameter

In order to present the obtained results quantitatively, the number of laser shots required to reduce the extinction at the irradiation wavelength to 1/e of its initial value was calculated (No._1/e_) (Fig. 11). All the data points were fitted with the exponential decay function, which allowed to designate the No._1/e_ parameters for individual samples. Irradiation of the nanoprisms with the laser power of 920 mW at the wavelength of 800 nm required 6000 and 7500 of the laser shots for the B-GNPs and Ch-GNPs, respectively, to reach the established extinction intensities. Change of the laser power to 30 mW significantly increased the stability of both types of nanoprisms. Under resonance conditions, the No._1/e_ parameters were as follows: 76,000 and 245,000 of the laser shots for the B-GNPs and Ch-GNPs, respectively. The summary of data about the melting experiment is shown in Table 1.

**Table 1:** Summary of the melting experiment

Sample name	Irradiation wavelength	Laser power	Laser fluence	No.1/e (×10^3^)	Total decline of triangular nanoparticles	GNPs precipitating from the solution
B-GNPs	800 nm	920 mW	3.2 mJ/cm^2^	6	Yes	No
	800 nm	30 mW	0.11 mJ/cm^2^	76	No	No
Ch-GNPs	800 nm	920 mW	3.2 mJ/cm^2^	7.5	No	Yes
	650 nm	30 mW	0.11 mJ/cm^2^	245	No	Yes

Presented results indicate that the chemically synthesized gold nanoprisms are more stable during the laser irradiation, irrespective of the irradiation wavelength and the laser power. This may be related with the variation in the dimensions of the nanoprisms. The biologically synthesized gold nanoprisms are bigger and thinner, which may influence their higher tendency to the deformation. In contrast, more consistent structure of chemically obtained nanotriangles may ensure their stability. Moreover, the stabilizing agents present at the surface of formed nanoprisms may play a role, because plant components (such as proteins) present at the surface of biological GNPs may be more sensitive to the irradiation than the surfactant covering chemical nanoprisms. However, long-time irradiated chemical gold nanotriangles become unstable and precipitated from the solution. This is likely to be caused by the disruption of the surfactant-nanoparticle interactions and aggregation of the GNPs. A similar experiment was performed by El-Sayed’s group for the gold nanoprisms in periodic monolayer arrays prepared with nanosphere lithography technique (Huang et al. 2005). Applying the irradiation wavelength coinciding with the SPR band of the prismatic nanoparticles, they needed higher laser densities (3 min of irradiation with laser density of ≥ 4 W/cm^2^ under resonance conditions), than used in our experiments, to melt the GNPs. Referring to these results, we can conclude that solutions of both biological and chemical triangular nanoparticles are more sensitive to the laser irradiation than the nanotriangles deposited on glass slides.

## Conclusions

In the present work, we compared the photostability of gold nanoprisms obtained using biological and chemical synthesis. Irradiation of the gold nanoparticles with the femtosecond laser highly influences their morphology and stability. Induced changes can be easily followed by the UV-Vis absorption spectroscopy and transmission electron microscopy. Both biological and chemical triangular GNPs undergo deformations, when exposed to the laser beam at the wavelength placed within their SPR band. Even relatively low-energy pulses cause rounding of the tips and decrease of the extinction at the wavelength corresponding to the irradiation wavelength. Nevertheless, the shape of the chemical nanoprisms was found to be less susceptible to the laser beam and no fully melted (perfectly rounded) structures were observed. Moreover, the mismatch between the SPR band of these GNPs and the irradiation wavelength reduced the influence of the laser beam on their morphology. However, the water stability of the irradiated chemical nanoprisms was disrupted and decrease of their concentration in the solution was observed.

## References

[CR1] Chang S-S, Shih C-W, Chen C-D, Lai W-C, Wang CRC (1999). The shape transition of gold nanorods. Langmuir.

[CR2] Gonzalez-Rubio G, Guerrero-Martinez A, Liz-Marzan LM (2016). Reshaping, fragmentation, and assembly of gold nanoparticles assisted by pulse lasers. Acc Chem Res.

[CR3] Gordel M, Olesiak-Banska J, Matczyszyn K, Nogues C, Buckle M, Samoc M (2014). Post-synthesis reshaping of gold nanorods using a femtosecond laser. Phys Chem Chem Phys.

[CR4] Hashimoto S, Werner D, Uwada T (2012). Studies on the interaction of pulsed lasers with plasmonic gold nanoparticles toward light manipulation, heat management, and nanofabrication. J Photochem Photobiol C.

[CR5] Huang W, Qian W, El-Sayed MA (2005). Photothermal reshaping of prismatic Au nanoparticles in periodic monolayer arrays by femtosecond laser pulses. J Appl Phys.

[CR6] Huang X, Jain PK, El-Sayed IH, El-Sayed MA (2007). Plasmonic photothermal therapy (PPTT) using gold nanoparticles. Lasers Med Sci.

[CR7] Klekotko M, Matczyszyn K, Siednienko J, Olesiak-Banska J, Pawlik K, Samoc M (2015). Bio-mediated synthesis, characterization and cytotoxicity of gold nanoparticles. Phys Chem Chem Phys.

[CR8] Link S, Burda C, Nikoobakht B, El-Sayed MA (2000). Laser-induced shape changes of colloidal gold nanorods using femtosecond and nanosecond laser pulses. J Phys Chem B.

[CR9] Liu J (2006). Electrochemical fabrication of single-crystalline and polycrystalline Au nanowires: the influence of deposition parameters. Nanotechnology.

[CR10] Liu D (2015). Rapid synthesis of monodisperse Au nanospheres through a laser irradiation-induced shape conversion, self-assembly and their electromagnetic coupling SERS enhancement. Sci Rep.

[CR11] Lou Z, Kim S, Zhang P, Shi X, Fujitsuka M, Majima T (2017). In situ observation of single Au triangular nanoprism etching to various shapes for plasmonic photocatalytic hydrogen generation. ACS Nano.

[CR12] Olesiak-Banska J, Gordel M, Matczyszyn K, Shynkar V, Zyss J, Samoc M (2013). Gold nanorods as multifunctional probes in a liquid crystalline DNA matrix. Nano.

[CR13] Parinda V, Rahul S, Mamraj S, Aditya KD, Jayashree AD, Deepak M (2014). Generation of stable colloidal gold nanoparticles by ultrashort laser-induced melting and fragmentation. Mater Res Express.

[CR14] Polte J, Ahner TT, Delissen F, Sokolov S, Emmerling F, Thunemann AF, Kraehnert R (2010). Mechanism of gold nanoparticle formation in the classical citrate synthesis method derived from coupled in situ XANES and SAXS evaluation. J Am Chem Soc.

[CR15] Pustovalov VK (2016). Light-to-heat conversion and heating of single nanoparticles, their assemblies, and the surrounding medium under laser pulses. RSC Adv.

[CR16] Sau TK, Murphy CJ (2004). Seeded high yield synthesis of short Au nanorods in aqueous solution. Langmuir.

[CR17] Scarabelli L, Coronado-Puchau M, Giner-Casares JJ, Langer J, Liz-Marzan LM (2014). Monodisperse gold nanotriangles: size control, large-scale self-assembly, and performance in surface-enhanced Raman scattering. ACS Nano.

[CR18] Thiele M (2016). Gold nanocubes—direct comparison of synthesis approaches reveals the need for a microfluidic synthesis setup for a high reproducibility. Chem Eng J.

[CR19] van der Zande BMI, Böhmer MR, Fokkink LGJ, Schönenberger C (2000). Colloidal dispersions of gold rods: synthesis and optical properties. Langmuir.

[CR20] Wang H, Huff TB, Zweifel DA, He W, Low PS, Wei A, Cheng JX (2005). In vitro and in vivo two-photon luminescence imaging of single gold nanorods. Proc Natl Acad Sci U S A.

[CR21] Warshavski O, Minai L, Bisker G, Yelin D (2011). Effect of single femtosecond pulses on gold nanoparticles. J Phys Chem C.

[CR22] Yuan H, Khoury CG, Hwang H, Wilson CM, Grant GA, Vo-Dinh T (2012). Gold nanostars: surfactant-free synthesis, 3D modelling, and two-photon photoluminescence imaging. Nanotechnology.

[CR23] Zhao Y, Cao M, McClelland JF, Shao Z, Lu M (2016). A photoacoustic immunoassay for biomarker detection. Biosens Bioelectron.

[CR24] Zhou J, Ralston J, Sedev R, Beattie DA (2009). Functionalized gold nanoparticles: synthesis, structure and colloid stability. J Colloid Interface Sci.

[CR25] Zijlstra P, Chon JW, Gu M (2009). White light scattering spectroscopy and electron microscopy of laser induced melting in single gold nanorods. Phys Chem Chem Phys.

